# Dynamic changes in B cell subpopulations in response to triple-negative breast cancer development

**DOI:** 10.1038/s41598-024-60243-y

**Published:** 2024-05-21

**Authors:** Igor Visconte Gonçalves, Natália Pinheiro-Rosa, Lícia Torres, Mariana de Almeida Oliveira, Gabriela Rapozo Guimarães, Christiana da Silva Leite, José Miguel Ortega, Miriam Teresa Paz Lopes, Ana Maria Caetano Faria, Mariana Lima Boroni Martins, Liza Figueiredo Felicori

**Affiliations:** 1 Department of Biochemistry and Immunology, Universidade Federal de Minas Gerais, Av. Pres. Antônio Carlos, 6627 – Pampulha, Belo Horizonte, MG 31270-901 Brazil; 2 NYU Grossman School of Medicine, NYU Langone Health, New York University, 550 1st Ave, New York, NY 10016 USA; 3 Department of Pharmacology, Universidade Federal de Minas Gerais, Av. Pres. Antônio Carlos, 6627 – Pampulha, Belo Horizonte, MG 31270-901 Brazil; 4 Instituto Nacional de Câncer, Ministério da Saúde, Coordenação de Pesquisa, Laboratório de Bioinformática e Biologia Computacional - Rua André Cavalcanti, 37, 1 Andar, Centro, Rio de Janeiro, RJ 20231050 Brasil

**Keywords:** Triple-negative breast cancer, B LAG-3, B-TIL, Humoral immune response, Tumor-infiltrating lymphocytes, 4T1, E0771, Immunology, Adaptive immunity, Cellular immunity, Molecular medicine, Computational biology and bioinformatics, Biochemical reaction networks, Cellular signalling networks

## Abstract

Despite presenting a worse prognosis and being associated with highly aggressive tumors, triple-negative breast cancer (TNBC) is characterized by the higher frequency of tumor-infiltrating lymphocytes, which have been implicated in better overall survival and response to therapy. Though recent studies have reported the capacity of B lymphocytes to recognize overly-expressed normal proteins, and tumor-associated antigens, how tumor development potentially modifies B cell response is yet to be elucidated. Our findings reveal distinct effects of 4T1 and E0771 murine tumor development on B cells in secondary lymphoid organs. Notably, we observe a significant expansion of total B cells and plasma cells in the tumor-draining lymph nodes (tDLNs) as early as 7 days after tumor challenge in both murine models, whereas changes in the spleen are less pronounced. Surprisingly, within the tumor microenvironment (TME) of both models, we detect distinct B cell subpopulations, but tumor development does not appear to cause major alterations in their frequency over time. Furthermore, our investigation into B cell regulatory phenotypes highlights that the B10 Breg phenotype remains unaffected in the evaluated tissues. Most importantly, we identified an increase in CD19 + LAG-3 + cells in tDLNs of both murine models. Interestingly, although CD19 + LAG-3 + cells represent a minor subset of total B cells (< 3%) in all evaluated tissues, most of these cells exhibit elevated expression of IgD, suggesting that LAG-3 may serve as an activation marker for B cells. Corroborating with these findings, we detected distinct cell cycle and proliferation genes alongside LAG-3 analyzing scRNA-Seq data from a cohort of TNBC patients. More importantly, our study suggests that the presence of LAG-3 B cells in breast tumors could be associated with a good prognosis, as patients with higher levels of LAG-3 B cell transcripts had a longer progression-free interval (PFI). This novel insight could pave the way for targeted therapies that harness the unique properties of LAG-3 + B cells, potentially offering new avenues for improving patient outcomes in TNBC. Further research is warranted to unravel the mechanistic pathways of these cells and to validate their prognostic value in larger, diverse patient cohorts.

## Introduction

Despite comprising a minority of breast cancer diagnoses, triple-negative breast cancer (TNBC) is considered one of the worse types, as these tumors lack expression of estrogen (ER), progesterone (PR) and human epidermal growth factor receptor 2 (HER2), limited therapeutic options are available, and patients are often treated with to chemo- and radiotherapy^1^. Although affecting younger women and characterized by highly aggressive tumors, in recent years, it has been demonstrated that triple-negative tumors have a more pronounced immune response compared to the other types of the disease, presenting a higher frequency of tumor-infiltrating lymphocytes (TILs)^2,3^. Albeit standardization of TIL evaluation is still debated, distinct researchers observed better overall survival (OS) among patients with elevated lymphocytic infiltration. Moreover, higher TIL proportions in breast tumors have predicted a better response to anthracycline/taxane-based chemotherapies in both neoadjuvant and adjuvant therapy settings^4–6^, therapeutic regimens that are frequently implemented in Brazil for the treatment of breast cancer patients^7^.

Although promising results have emerged from recent studies, TIL encompasses distinct cell populations, and further characterization of the individual components is required for a better understanding of the disease dynamic, as well as their individual impact on carcinogenesis^5^. Despite the elevated heterogeneity in triple-negative tumors, studies demonstrate that TCD4 and TCD8 cells are the predominant lymphocyte population in the tumor microenvironment, usually found in direct contact with tumor cells or residing in stromal areas surrounding cancer cell islands^2,8^. Authors demonstrate that higher infiltration of T cells is associated with a good prognosis, showing the cooperative action between CD4 and CD8 phenotypes in controlling tumor expansion^9–11^. Additionally, TCD4-secreted cytokines (IFN-y, TNF-α and IL-2) majorly contribute to the activation of macrophages, dendritic, and NK cells, which collectively enhances anti-tumoral immune response^12^. Although higher lymphocyte infiltration is a positive finding, pro-tumoral Treg profiles are also detected in the tumor microenvironment (TME), and can significantly contribute to dampening the immune response through different mechanisms^13^. It’s been demonstrated that these cells can impair the activation of antigen-presenting cells through immune checkpoint signaling (TIM-3, VISTA, CTLA-4), prevent activation and proliferation of effector T cells, as well as secreting immune suppressive cytokines (IL-10, IL-35, TGF-β). Additionally, it’s been established that Tregs can promote neovasculature formation, which contributes to tumor expansion and establishment of metastasis^14,15^.

While major progress has been achieved in understanding the role of distinct T cell subpopulations, studies demonstrate that B cells can also be found in the TME. Albeit capable of recognizing mutated proteins and producing antibodies against tumor-associated antigens^16^, the overall impact of B lymphocytes on breast tumors is still debated. Although conducted in different clinical settings, authors emphasize the importance of B cells in controlling tumor development, showing a longer disease-free survival (DFS) in patients presenting higher levels of TIL-Bs, was well as elevated CD20 in sentinel lymph nodes^17–19^. Additionally, in transcriptome data analysis, B-cell signatures in breast tumor samples have been associated with improved survival^19^. Oppositely, others argue that B cells can contribute to tumor progression. One study demonstrated a positive association between the density of B cells and high histological-grade tumors^20^. Moreover, other studies showed a shorter recurrence-free survival (RFS) in patients with B-cell and antibody-secreting cells (ASCs) infiltrates^21,22^.

Intriguingly, in recent years some authors have proposed that regulatory B cells (Bregs) may also contribute to the disease progression^23^. Early studies demonstrate that Bregs can emerge from distinct B cell subsets and are capable of inhibiting other immune cell and pro-inflammatory pathways^24,25^. Although B10 cells comprise one of the mainly studied Bregs, other populations with distinct surface markers have been revealed^26^. Known for its homology to the CD4 receptor, LAG-3 is usually expressed on early-activated T cells and FoxP3 + Tregs, and upon interaction with its ligand – mostly MHC-II molecules, it inhibits the activation of the targeted cell^27,28^. Interestingly, Lino and colleagues^29^ identified a subset of plasma cells expressing LAG-3 and elevated IL-10 expression. Despite the production of IL-10, IL-35, and TGF-β being proposed to be the main mechanism of immune suppression, others suggest that Bregs can promote the differentiation of effector T cells in Tregs, as well as affect TNF-α production in monocytes^30,31^. Although detection of these regulatory profiles has been associated with worse prognosis in gastric and hepatocellular carcinomas^32,33^, the impact of these populations remains to be evaluated in breast tumors. While distinct Breg phenotypes have been proposed, the lack of cell-specific transcriptional factors, as well as differences between mice and humans poses a challenge in understanding this potential new B cell subpopulation^31^.

Despite the promising emerging results, the dynamic of tumor-infiltrating B lymphocytes remains a challenge, as these populations are presented in lower frequency and are laborious to retrieve. Furthermore, most studies are conducted in advanced tumor or treatment settings, making it difficult to understand the impact of earlier carcinogenic events in these cells. Considering the capacity of tumor cells to modulate the immune response in the tumor microenvironment and relatively sparse literature about B cells in breast tumors, we assessed the impact of 4T1 and E0771 murine breast tumor development on the B cell compartment and regulatory surface markers in secondary lymphoid organs and the TME. Additionally, we extend our research to validate the occurrence of rare B cell subsets within a human patient cohort and examine their correlation with patient survival outcomes.

## Materials and methods

### Cell lines

4T1 (ATCC, CRL- 2539) and E0771 (CH3 Biosystems, 94001) cell lines were kindly donated by Dra. Miriam Lopes and Dra. Catarina Raposo Dias Carneiro from the Institute of Biological Sciences at the Federal University of Minas Gerais/MG, and the Faculty of Pharmaceutical Sciences at the. State University of Campinas/SP, respectively. 4T1 and E0771 cell lines were cultured in RPMI-1640 medium (Gibco) supplemented with 10% Fetal Bovine Serum and 1% Streptomycin/Penicillin (Gibco), and kept at 37 °C and 5% CO_2_ incubator. The cell culture medium was replenished every two or three days, and cell lines were subcultured when cells achieved 70% confluence.

### Animals

Wild-type BALB/c and C57BL/6 mice (8 to 10 weeks old female) were obtained from Central Bioterium (ICB/UFMG). The mice were housed under specific pathogen-free conditions at the Laboratory of Immunology (ICB/UFMG), with free access to food and water. Approval for all studies was granted by the Ethics Committee on Animal Use at the Comissão de Ética no Uso de Animais da UFMG (CEUA-UFMG)/Brazil, under protocol number 215/2018. All methods were performed in accordance with the Committee and relevant guidelines and regulations, including the ARRIVE guidelines (https://arriveguidelines.org).

### Breast cancer model

*Mycoplasma-free* 4T1 and E0771 tumor cells were resuspended in sterile phosphate-buffered saline (PBS) (Invitrogen), and 5 × 10^5^ cells were injected (s.c.) in the mammary fat pad of BALB/c and C57BL/6 female mice, respectively. As for the control group, mice received an injection (s.c.) of sterile PBS. After initial inoculation, mice were monitored twice a week for tumor development. After achieving the corresponding period of analysis (7, 14, and 21 days after the tumoral challenge), mice were euthanized, and the spleen, inguinal lymph nodes, and tumor were surgically removed and processed for further analysis. Tumors were measured with a digital caliper. Tumor volume (V) was calculated based on the following formula: V = (W)^2^ × L × 3.14/6; W is width, and L is length. Spleen and tDLN organ area was calculated using ImageJ, and the fold change was calculated as it follows: (Tumor group average organ area $\div$ Control group average organ area). Lymph nodes, spleen, and tumors were weighted and the area was calculated using ImageJ software. Each experiment was performed twice. tDLN/Spleen sample number (n = 5 to 6), Tumor sample number (total n = 9, each datapoint represents a pool of 3).

### Flow cytometry

Surgically excised tumors were minced using scissors in non-supplemented RPMI media on ice. Tumors pieces were resuspended in 4 mL of RPMI medium and transferred to a 15-mL conical tube containing 3 mL of Ficoll-Paque 1.084 and subsequently centrifuged for 40 min at RT. Cells were washed twice with 10 mL 1X PBS to remove potential contaminants. Tumor single cells were suspended in 0.5 mL of 1X PBS. Spleen and tDLN single-cell suspensions were obtained by grinding the tissue against a well of a 24-well plate with a syringe thumb rest on ice. Cells were transferred to a 15-mL conical tube containing 5 mL of RPMI medium and centrifuged at 1500 RPM for 5 min at 4 °C. tDLN samples were resuspended in 0.5 mL PBS, while spleen samples were vigorously resuspended in 9 mL of distilled water for 10 s, followed by the addition of 1 mL of 10X PBS for red blood cell lysis, adapted method from previous work^34^. After centrifugation at 1500 RPM for 5 min at 4 °C, spleen samples were suspended in 1 mL of 1X PBS. After dead cells assessment by trypan blue cell viability assay, 1 × 10^6^ cells were added to a 96-well plate, centrifuged (1500 RPM, 4 °C), and incubated in viability dye and Fc blocker (1:1000, 0.01 µg/µL in PBS, respectively) for 30 min at RT. Cells were centrifuged, washed, and incubated with antibodies according to the manufacturer’s instructions. Cells were acquired in FACSFortessa and all analysis were conducted in FlowJo v.10 software. Cellular populations were evaluated through two distinct flow cytometry panels (PN), with the first one used to asses myeloid and lymphoid cells, while the second panel was focused on analyzing the B cell compartment. Correspondent gating strategy for each panel is described as followed. PN1 (Supplementary Fig. S1a): Total cells selected based on FSC-A and SSC-A > selection of single cells > total live leukocytes CD45 + . For myeloid cell subpopulations: total myeloid cell based on CD11b + expression and SSC-H > discrimination of mononuclear (Ly-6C + Ly-6G-) and polymorphonuclear (Ly-6Clow- Ly-6G +). For lymphoid cell subpopulations: discrimination of B (CD19 +) and T (CD3 +) lymphocytes > discrimination of cytotoxic (TCD8 +) and helper (CD4 +) T cells. PN2 (Supplementary Fig. S1a): Total cells selected based on FSC-A and SSC-A > selection of single cells > total live leukocytes CD45 + . For plasma cells: CD138 + and CD19^+/–^ expression. For Pro-B10 B cells: CD19 + CD5 + CD1d + . For Naive and early-activated B cells: CD19 + IgD^high^, and CD19 + IgD^int-low^. For LAG-3 + B cells: CD19 + LAG-3 + . For Naive and early-activated LAG-3 + B cells: CD19 + LAG-3 + IgD^high^, and CD19 + LAG-3 + IgD^int-low^. For LAG-3 plasma cells: LAG-3 + CD138 + . All associated fluorochromes are described in Table 1.

**Table 1:** Antibodies employed for flow cytometry analysis in this study*.

Reagent or resource	Dilution	Source	Identifier
Antibodies			
Brilliant Violet 421™ anti-mouse CD4	1:200/PN1	Biolegend	100437
FITC anti-mouse CD45	1:300/PN1	Biolegend	103108
PE-CF594 Hamster anti-mouse CD3e	1:200/PN1	BD	562332
PE-Cy™7 Rat anti-mouse Ly-6C	1:200/PN1	BD	560593
PE Rat anti-mouse CD19	1:300/PN1	BD	557399
PerCP-Cy™5.5 Rat anti-CD11b	1:300/PN1	BD	561114
APC anti-mouse CD8a	1:100/PN1	Biolegend	100712
APC-Cy™7 anti-mouse Ly-6G	1:200/PN1	Biolegend	127624
Pacific Blue™ anti-mouse CD1d	1:100/PN2	Biolegend	123517
FITC Rat anti-mouse CD5	1:100/PN2	BD	553021
PE-Dazzle™594 anti-mouse CD223 (LAG-3)	1:100/PN2	Biolegend	125224
PE-Cy™7 anti-mouse CD138	1:100/PN2	Biolegend	142514
PE anti-mouse IgD	1:300/PN2	Biolegend	405706
PE-Cy™5 Rat anti-mouse CD19	1:300/PN2	Invitrogen	15-0193-81
Alexa Fluor^®^ 700 Rat anti-mouse CD45	1:300/PN2	BD	560510
LIVE/DEAD Fixable Yellow Dead Cell Stain Kit	–	Invitrogen	L34959

*The tDLNs and spleen from E0771 and 4T1 tumor models were evaluated using two distinct flow cytometry panels (PN1, PN2). Tumor samples were assessed only through panel II due to sample restriction. All antibodies were diluted in a cooled PBS solution.

### Single-cell RNA-seq data acquisition

To better understand tumor-infiltrating B cell subpopulations, clinical and scRNA-seq integrated data, yet unpublished, were shared by the Bioinformatics and Computational Biology Laboratory of the Brazilian National Cancer Institute/RJ. scRNA-Seq data comprising 392,204 high-quality cells derived from 13 public dataset, including distinct technologies (10 × Genomics, InDrop, and Smart-Seq2) was initially used for the analysis. Cells identified as B lymphocytes (21.947 single cells) (expressing CD79A/B, MS4A1, and MZB1) were selected. Subsequently, 3000 highly variable genes were calculated, with batch effect correction for each sample, followed by integration using the scVI algorithm^35^ from the scvi-tools library (v1.1.0)^36^. The latent space generated by scVI was projected into a 2D space using the UMAP^37^. Clustering was performed using the Leiden algorithm^38^ in a range of values from 0.3 to 1. The Leiden resolution analyzed was set to 0.7, and manual annotation was performed by observing canonical markers and their relation to differentially expressed genes calculated by the Model-based Analysis of Single-Cell Transcriptomics (MAST) algorithm^39^ using the FindAllMarkers() function of Seurat (v 4.3.0)^40^.

### Digital cytometry and survival analysis

To investigate the clinical impact of different B LAG3^+^, we downloaded bulk RNA-Seq data and clinicopathological data from the Breast Cancer (BRCA) cohort of The Cancer Genome Atlas (TCGA) database using the TCGAbiolinks package (v.3.17)^41^. Subsequently, we estimated the proportions of the cellular subtypes identified in the analysis in these bulk RNA-Seq samples through a deconvolution approach using the BayesPrism digital cytometry tool (v2.1)^42^. We considered 45 cellular signatures, combining the subpopulations described in the manuscript^1^—including fibroblasts, endothelial cells, mast cells, plasmacytoid dendritic cells, malignant cells, normal breast cells, macrophages (n = 12), monocytes (n = 6), conventional dendritic cells (n = 8), T cells (n = 9), NK cells (n = 2), neutrophils (n = 3), and excluding those were described with a proliferative profile—and the B lymphocytes (n = 5) characterized here. Genes expressed in fewer than 5 cells, ribosomal genes, mitochondrial genes, X and Y chromosome-linked genes, and the MALAT gene were removed using the *cleanup.genes()* function. Afterward, to obtain more consistent results, we filtered protein-coding genes and used the *get.exp.stat()* function to select differentially expressed genes between cellular states of different cell types using default parameters, except for the pseudo-count parameter set to 0.1, as the data were predominantly from 10x. The default parameters of the *new.prism()* function was applied, using tumor cells as a reference. Subsequently, to assess the impact of all cell types on survival, we calculated the hazard ratio (HR) of the relative fractions of each cell type population divided into quartiles, with 95% confidence intervals (CI) based on maximum likelihood estimates for each covariate using the univariate Cox regression model (p < 0.05). The Survival (v3.5) and Survminer (v0.4.9) packages^43,44^ were employed for these analyses. The *surv_cutpoint()* function from the Survminer package was applied to divide samples into High and Low groups based on the enrichment of B lymphocyte subpopulations among TCGA patients. Subsequently, to assess the impact of the LAG-3 + B cells on progression-free interval (PFI) and overall survival (OS), we applied the omnibus MDIR test, which combines the log-rank, Mantel Haenszel, and Tarone-Ware tests for non-proportional hazards in the survival curves of the Kaplan–Meier.

### Statistical analysis

Numeric data were presented as mean ± standard deviation. Normally distributed data were analyzed by Student *t*-test (two groups) or one-way ANOVA (more than two groups) with the Tukey test. Non-normal distributed data were analyzed by the Mann–Whitney rank test (two groups), Kruskal–Wallis test (more than two groups) with Dunn’s test. A two-tailed p-value of < 0.05 was considered statistical significance. All statistical analyses were performed using GraphPad Prism 8.0 for Macintosh (GraphPad Software, San Diego, CA, USA).

## Results

### 4T1 tumor elicits significant morphologic alterations in the tumor microenvironment and secondary lymphoid organs

**Figure 1:**
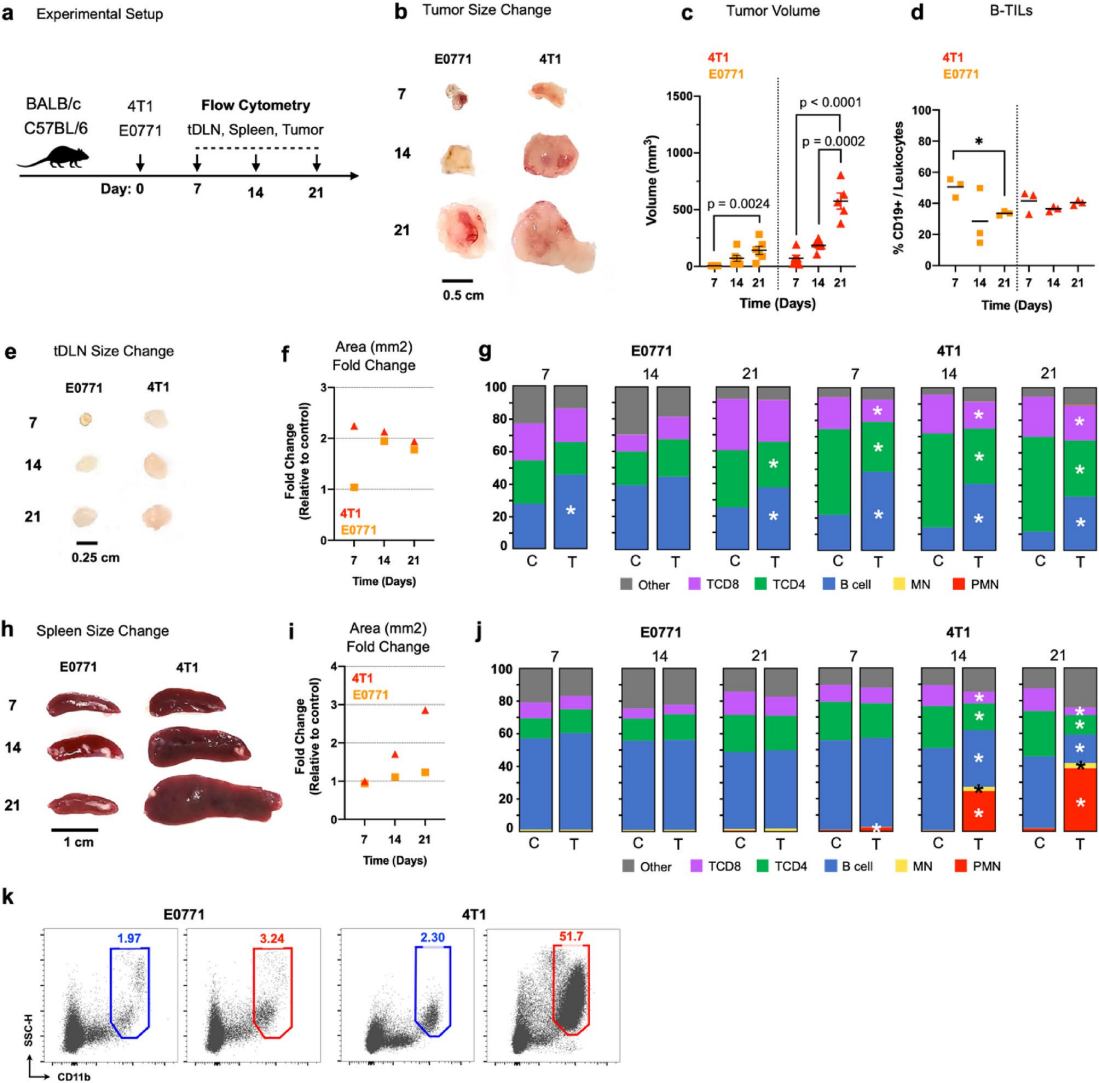
Impact of tumor development in morphologic and cellular composition in the TME and SLOs. 8-week-old female mice were assigned to control and tumor groups. BALB/c and C57BL/6 mice were injected (s.c.) with 5 × 10^5^ 4T1 and E0771 tumor cells, respectively. Mice from the control group were injected (s.c.) with an equivalent volume of sterile PBS solution. Cell lines were tested for mycoplasma sp. contamination before inoculation. Mice were euthanized on days 7, 14, and 21, and the tumor-draining lymph node (tDLN), spleen, and tumor were surgically excised and processed for flow cytometry analysis (**a**). Representative images of E0771 and 4T1 tumors (**b**). Tumor volume (mm^3^) for 4T1 and E0771 tumors throughout the weeks (**c**). Frequency of tumor-infiltrating B cells (B-TILs). Tumor samples were processed mechanically, and mononuclear TILs isolated through Ficoll-Paque centrifugation. Each dot represents a pool of 3 tumors (total n = 9) (**d**). Representative images of the tDLNs from E0771 and 4T1 tumor groups (**e**). Area (mm^2^) fold change of the tDLNs between control and tumor group mice for 4T1 and E0771 models (**f**). Frequency (as % of total CD45 +) of TCD8, TCD4, B cells, mononuclear myeloid cells (MN), and polymorphonuclear myeloid cells (PMN) of the tDLNs (**g**), and spleen (**j**) for control (C) and tumor (T). Representative images of the spleen from E0771 and 4T1 tumor groups (**h**). Area (mm^2^) fold change of the spleen between control and tumor group mice for 4T1 and E0771 models (**i**). Gated CD11b + myeloid cells from spleen samples (21 Days) from control (blue) and tumor (red) groups for 4T1 and E0771 models (**k**). 4T1 (red), E0771 (orange). One-way ANOVA Tukey post hoc test (**c**,**d**). Student *t*-test (**g**,**j**). *p < 0.05, **p < 0.01, ***p < 0.001, ****p < 0.0001.

It’s well recognized that tumor development changes the dynamic of the immune response not only in the TME but also in lymphoid organs. Although most studies have been primarily focusing on understanding the kinetics of T cells, B cells are also observed in tumors, how cancer development affects the B cell compartment remains to be further elucidated. To determine the impact of breast cancer development on the B cell dynamic in the TME and secondary lymphoid tissues, we used two distinct in vivo murine breast tumor models. Syngeneic immunocompetent 8-week-old female BALB/c and C57BL/6 mice were distributed in tumor and control groups, and injected (s.c) in the mammary fat pad with 5 × 10^5^ 4T1 and E0771 tumor cells, respectively. Mice from the control group were injected (s.c) with an equivalent volume of PBS solution. Following initial inoculation, mice were followed weekly for tumor assessment and were euthanized after 7, 14, and 21 days (n = 6, each period of analysis). Spleen, tDLN, and tumors were surgically excised, measured, and processed for flow cytometry analysis (Fig. 1a).

Although both murine models presented palpable primary tumors after 7 days following initial inoculation, in each correspondent period of analysis, 4T1 tumors were larger compared to E0771 tumors (Fig. 1b). While the difference in tumor growth between the two murine models may involve distinct genetic and molecular pathways, 4T1 and E0771 cell lines present distinct morphologic features, with 4T1 cells presenting an epithelial-like polygonal shape, while E0771 cells displayed an elongated shape. Furthermore, in all timepoints 4T1 induced tumors were denser and presented more necrotic areas compared to E0771 induced tumors, which were softer (Supplementary Fig. 4). Additionally, 4T1 tumor development was more pronounced compared to E0771, as 4T1 tumor volume was considerably larger than E0771 tumors on day 21 (volume: 142 mm3 vs. 575 mm3) (Fig. 1c). Although both tumors presented a meaningful change in size and volume over time, among isolated infiltrating mononuclear cells, only E0771 tumors demonstrated alteration for B cells, featuring a decrease between the first and third week, while no changes were seen for 4T1 tumors (Fig. 1d). Interestingly, the impact of 4T1 and E0771 tumor growth was distinct in secondary lymphoid organs. Although both tumor models caused an increase of the tDLN, the enlargement of the organ was more noticeable in the 4T1 model, as early as the first week, while in the E0771 model, a pronounced fold change in the lymph node area was only seen from the second week on. Despite this initial difference, draining lymph nodes persisted larger throughout tumor development (Fig. 1e,f, Supplementary Fig. S2).

When investigating the cellular populations, it was demonstrated an elevation of B cell frequency in both murine models when compared to mice from the control group, yet this relative increase was more evident in 4T1 models through the weeks (Fig. 1g). Corroborating with these findings, we also observed a more robust elevation in the absolute cell count for B and T cells in the 4T1 model (Supplementary Fig. S3a–c). Although myeloid cells were detected in the tDLNs in both murine models, mono (MN) and polymorphonuclear (PMN) populations corresponded only to a minor fraction of total leukocytes (< 1%) (Supplementary Fig. S3d–e). Conversely, morphologic changes in the spleen were only detected for the 4T1 model (Fig. 1h), with a threefold change in organ area in the third week comparing mice from tumor and control groups (Fig. 1i). While major increment in CD11b + myeloid cells was observed in the spleen of 4T1 tumor-bearing mice throughout the weeks – comprising on average for almost half of the leukocytes in the last period of analysis (Fig. 1j,k), we did not detect any significant alterations for mice injected with E0771 tumor cells. Moreover, mononuclear myeloid cells in 4T1 tumor-bearing mice only accounted for < 5% of total leukocytes (Supplementary Fig. S3j), with a predominance of polymorphonuclear myeloid cells. Compared to 4T1 tumor-bearing mice, mice injected with E0771 cells presented a more pronounced increase in the absolute cell count for the splenic B and T cell populations (Supplementary Fig. S3f–h). Despite that, in opposition to tDLNs, the elevation of total B and T cells was only detected in the last period of analysis (21 days).

### tDLNs of 4T1 and E0771 models present expansion of distinct B cell subsets

**Figure 2:**
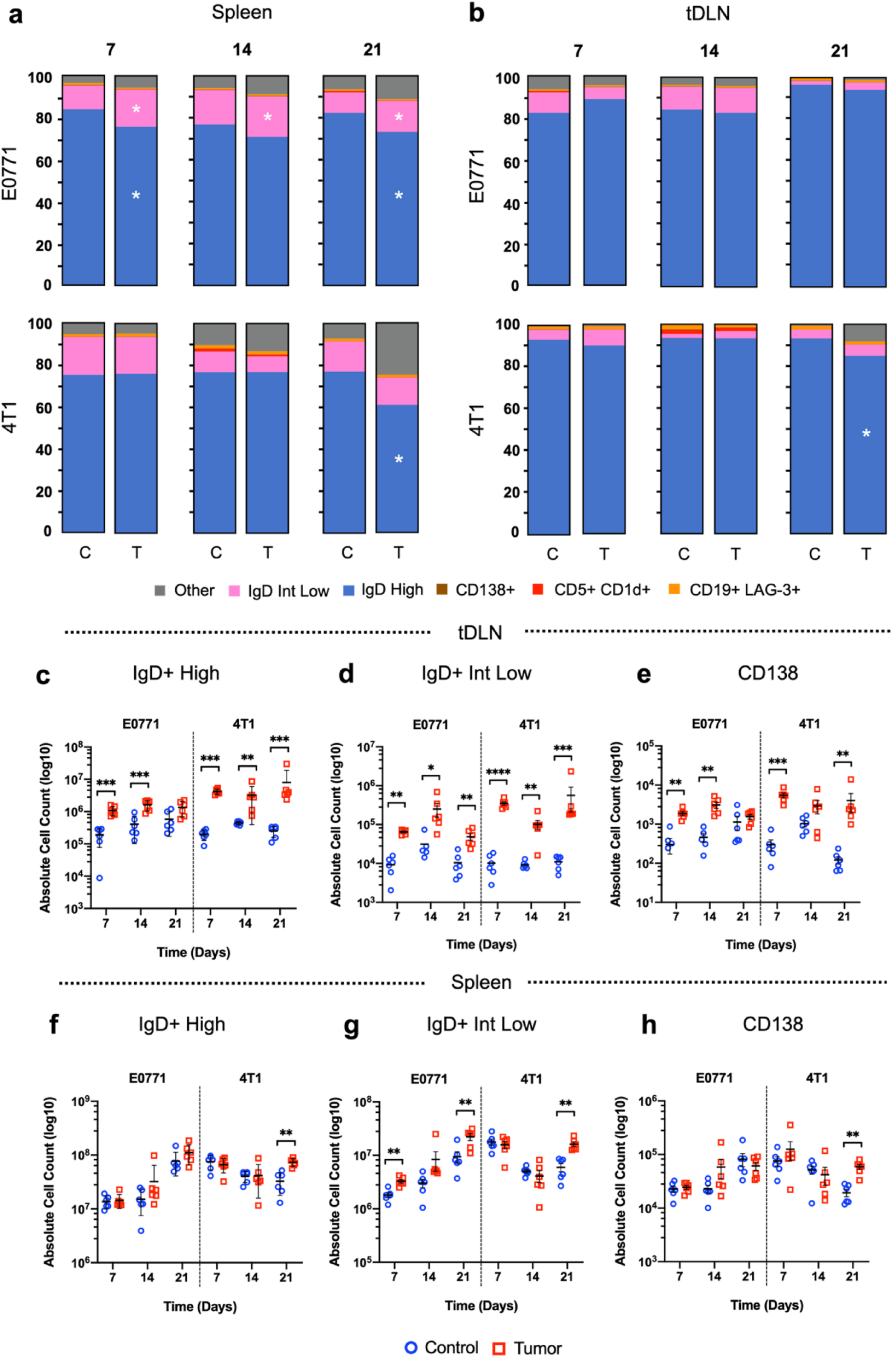
Evaluation B cell subsets in tDLN and spleen from E0771 and 4T1 tumor-bearing mice. Frequency of IgD + High (pink), IgD + intermediate/low (blue), CD138 + (brown), CD5 + CD1d + (red), CD19 + LAG-3 + (orange) as % of total B cells (CD19 +) in the spleen (**a**), and tDLNs (**b**) after 7, 14 and 21 days of tumor challenge with 4T1 and E0771 cells. Subcolumns C and T, as control and tumor groups. Absolute cell count of IgD + High (**c**,**f**), IgD + intermediate/low (**d**,**g**), and plasma-cells (**e**,**h**) for control and 4T1/E0771 tumor-bearing mice in the tDLNs and the spleen, respectively. Student *t*-test (**c**–**h**). p < 0.05, **p < 0.01, ***p < 0.001, ****p < 0.0001.

To further investigate the impact of tumor development in the B cell dynamic in the tumor microenvironment and associated lymphoid organs, we evaluated the distinct B cell subsets following tumor challenge. Although showing distinct tumor development kinetics and 4T1-induced tumors promoting splenomegaly and early elevation of the area in tDLNs, CD19 + IgD + high was the predominant subpopulation (CD19 + IgD +) in both spleen and tDLNs of control and 4T1 and E0771 tumor-bearing mice (Fig. 2a,b). Despite that, tDLNs presented a noticeable elevation of IgD + high phenotype, with a significant increase of this B cell subset as early as 7 days for both models (Fig. 2c). While the elevation of IgD + high cell phenotype is an indication of proliferating B cells upon antigen recognition, lower expression of this cell marker suggests an ongoing differentiation process. Evaluating IgD + intermediate/low population, we observed a significant increase of this phenotype in both models for tDLNs samples. Despite that, 4T1 presented a more robust elevation compared to the E0771 model (Fig. 2d). In addition to IgD + high and intermediate/low B cell profiles, we also noticed an elevation of plasma cells (CD138 +), further suggesting a sustained proliferation and differentiation process in tDLNs as early as 7 days of tumoral challenge (Fig. 2e). While significant changes were detected for tDLNs in both murine models, elevation was modest for the spleen, presenting an increase of IgD high, intermediate/low, and plasma cells (CD138 +) only after 21 days of tumoral challenge in the 4T1 murine model. (Fig. 2f–h).

**Figure 3:**
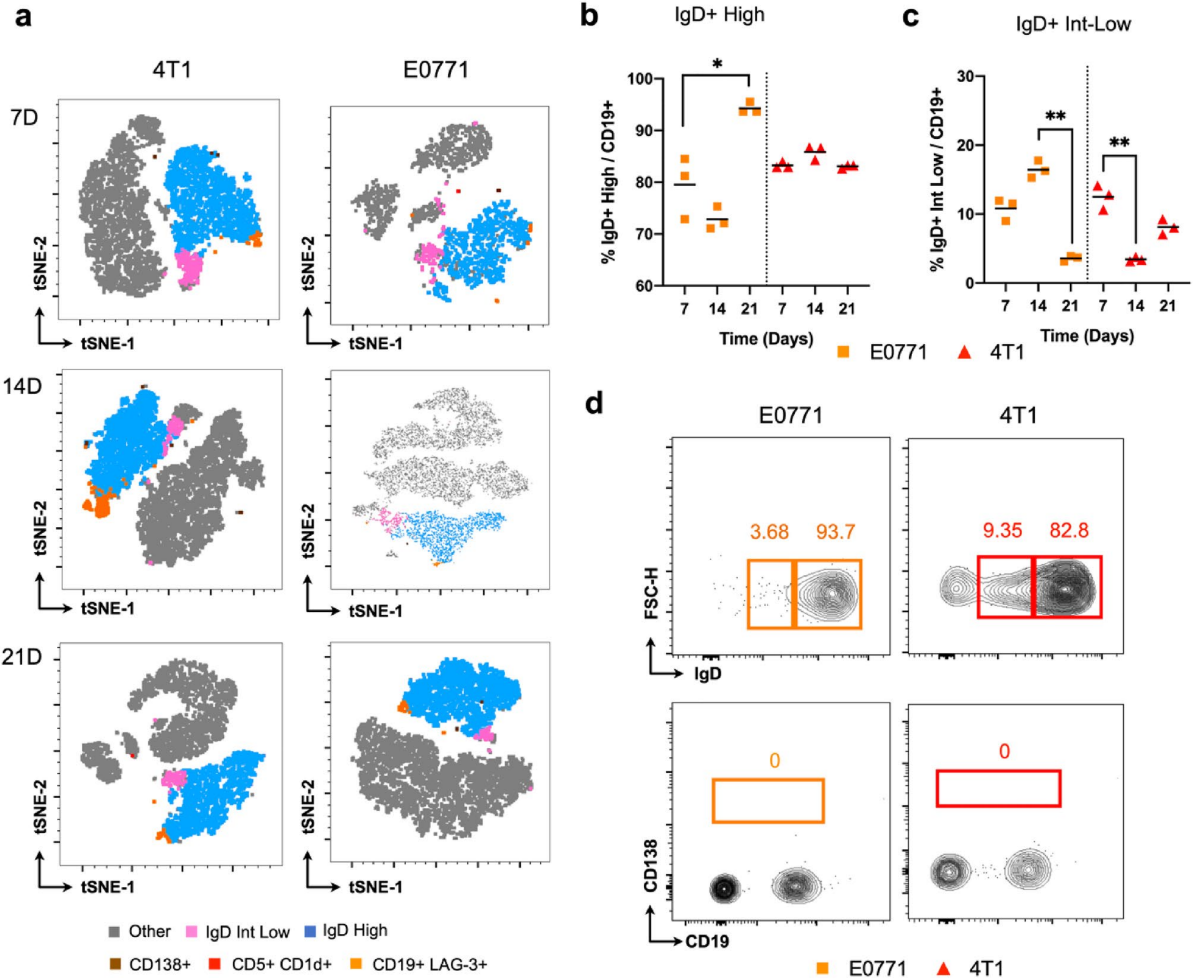
Evaluation B cell subsets in E0771 and 4T1 tumors. tSNE analysis depicting distinct B cell subsets through E0771 (right) and 4T1 (left) tumor development after 7, 14, and 21 days of initial inoculation (**a**). Frequency of IgD + high (**b**) and intermediate/low B cells (**c**) in 4T1 and E0771 tumors. Each dot represents a pool of 3 tumors (total n = 9). Gated IgD + high and intermediate/low B cells (top) and CD138 + cells (low) in E0771 (orange) and 4T1 (red) 21-day tumors (**d**). Kruskal–Wallis test, Dunn’s post hoc test (**b**,**c**). p < 0.05, **p < 0.01, ***p < 0.001, ****p < 0.0001.

Although the tumor microenvironment is characterized by immune suppression, B cells are still detected in the TME as previously demonstrated (Fig. 1d). Interestingly, analyzing B cell subsets in 4T1 and E0771 tumors we observed a similar profile to tDLNs and spleen, in which IgD + high was the predominating B cell subset, suggesting that although 4T1 and E0771 induced tumors have distinct kinetics profile, the humoral immune response in the TME is similar (Fig. 3a). Although we did not observe changes for this population in 4T1 tumors, we detected an elevation for E0771 between the first and third week of the tumoral challenge (Fig. 3b). Conversely, while in distinct period intervals, both 4T1 and E0771 had a decrease in IgD + intermediate/low population (Fig. 3c). While the presence of these subsets could indicate the proliferation and differentiation of B cells in the TME, cell events corresponding to plasma cells were rare or absent in both murine models (Fig. 3d), which could be explained by the lower cell yield in tumor samples compared to lymph nodes, as well as distinct differentiation process between the evaluated compartments.

### LAG-3 B cells in the TME and SLOs are predominantly IgD + 

**Figure 4:**
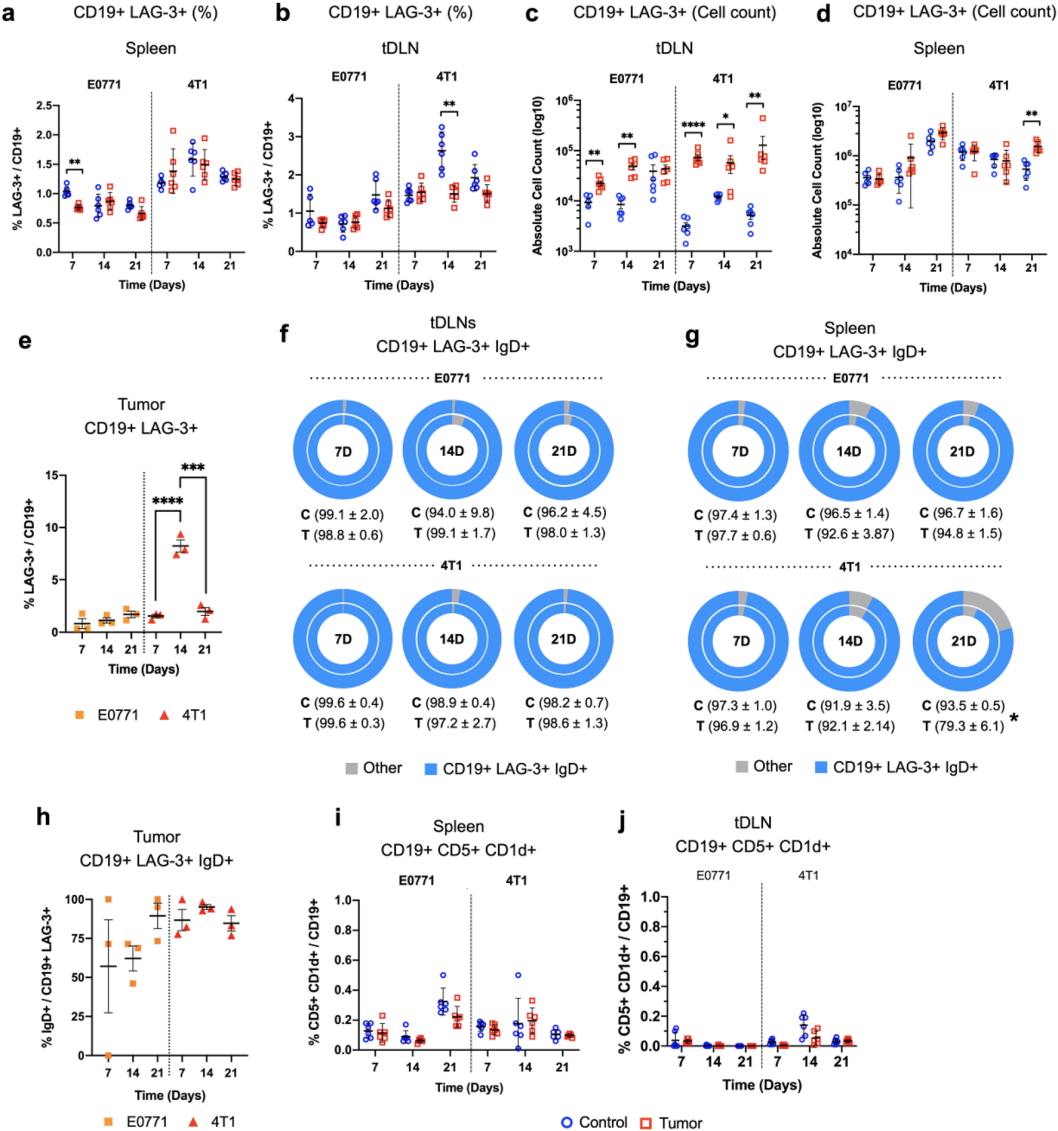
Evaluation of LAG-3 + and CD5 + CD1d + subsets among B-TILs in E0771 and 4T1 spleen, tDLNs and tumors. Frequency (%) of CD19 + LAG-3 + B cells in the spleen and tDLNs for control, and 4T1 and E0771 tumor groups, respectively (**a**,**b**). Absolute cell count of CD19 + LAG-3 + B cells in the tDLNs and spleen for control, and 4T1 and E0771 tumor groups, respectively (**c**,**d**). Frequency (%) of CD19 + LAG-3 + B cells in 4T1 (red) and E0771 (orange) tumors. Each dot represents a pool of 3 tumors (total n = 9) (**e**) Frequency (%) of CD19 + LAG-3 + IgD + B cells in the 4T1 and E0771 tumors. Each dot represents a pool of 3 tumors (total n = 9) (**f**). Frequency of CD19 + LAG-3 + IgD + in the spleen (**g**) and tDLN (**h**) of control (inner circle) and 4T1 and E0771 tumor-bearing mice (outer circle). Frequency of CD19 + CD5 + CD1d + cells in the spleen (**i**) and tDLNs (**j**). Control (blue), Tumor (red). Student T-test (**a**–**d**; **f**,**g**; **i**,**j**), Kruskal–Wallis test, Dunn’s post hoc test (**e**,**h**). *p < 0.05, **p < 0.01, ***p < 0.001, ****p < 0.0001.

Considering the capacity of tumor cells to promote regulatory phenotypes in the TME, and recent evidence demonstrating distinct phenotypes of Bregs, we evaluated the impact of tumor development in the expression of LAG-3 in B cells in SLOs and in the TME throughout time. In addition, we also evaluated the expression of CD5 and CD1d, surface markers that characterize the B10 Breg population. Although the function of LAG-3 on B cells still needs to be better elucidated, we observed the expression of the immune checkpoint only in a fraction of B cells for all evaluated tissues, with CD19 + LAG-3 + cells corresponding to 1–3% of total B cells in the tDLNs and the spleen (Fig. 4a,b). Curiously, despite the fact that the frequency of these cells remained relatively similar throughout time in the spleen and tDLNs, we observed a slight decrease for the subset in the first week for the E0771 model in the spleen, and in the second week for the 4T1 model in tDLNs (Fig. 4a,b). Despite that, the tDLNs from E0771 and 4T1 tumor-bearing mice presented an increased absolute cell count of CD19 + LAG3-3 + cells compared to the control group mice (Fig. 4c). Interestingly, although an elevation of the absolute cell counts for CD19 + LAG-3 + cells was also detected for the spleen, this elevation was restricted to the spleen after 21 days of tumoral challenge (Fig. 4d). As for tumor samples, similar to the tDLNs and spleen we observed that CD19 + LAG-3 + comprised only a fraction of total B cells. Although a transitory elevation in the frequency of this phenotype was observed for the 4T1 model 14 days after the initial inoculation, CD19 + LAG-3 + comprised on average 3% of total B cells in the TME of both murine models (Fig. 4e).

When analyzing distinct markers among LAG-3 + B cells, we observed that most of these cells in the spleen and tDLNs had IgD expression for both control and tumor-bearing mice, accounting for ≥ 90% of CD19 + LAG-3 + cells (Fig. 4f–g). Similar numbers were found for 4T1 tumors, in which we also reported elevated IgD expression among CD19 + LAG-3 + cells, while the frequency of the same phenotype was more variable for the E0771 tumor model (Fig. 4h). Although we couldn’t observe a significant change of CD19 + LAG-3 + IgD + cells for tDLNs and tumor samples in neither of the models throughout the weeks (Fig. 4f,h), we detected a decrease of the population in the spleen of 4T1 tumor-bearing mice 21 days after tumor inoculation (Fig. 4g).

Though considered one of the main Breg populations, and frequently found in the spleen and peritoneal cavity, CD5 + CD1d + B cells comprised only a minor portion of the evaluated phenotypes, comprising less than 1% of total B cells in the spleen of both murine models, in control and tumoral conditions (Fig. 4i). Moreover, although B10 B cells were also observed in the tDLN, the population only comprised a fraction of total B cells (< 0.5%), in which no major alterations were observed throughout 4T1 and E0771 tumor development (Fig. 4j).

### LAG-3 B cells are associated with a good prognosis in TNBC patients

**Figure 5:**
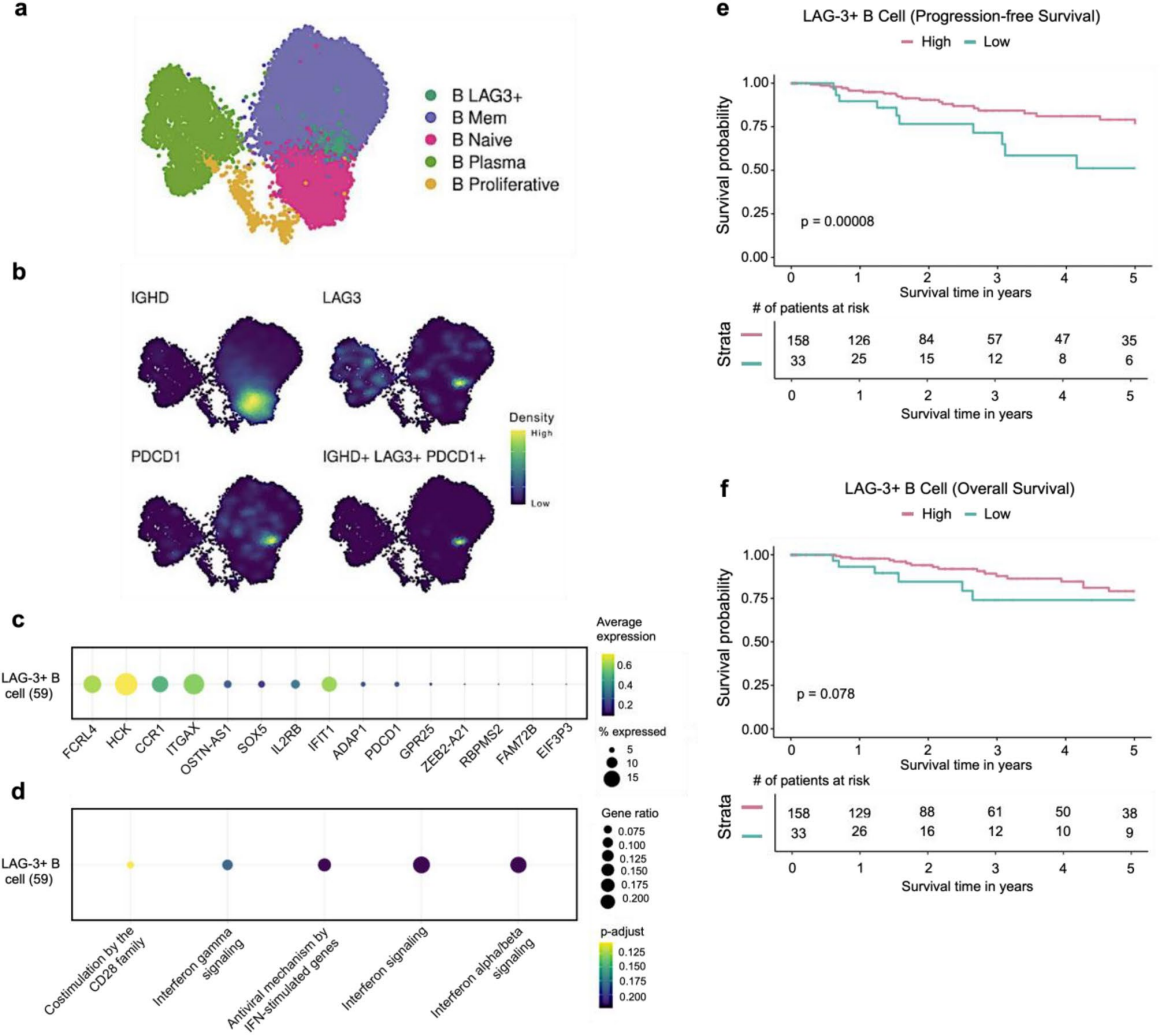
Analysis of tumor-infiltrating LAG-3 B cells in TNBC patients. (**a**) Uniform Manifold Approximation and Projection (UMAP) of tumor-infiltrating B cells, color-coded according to cell types. (**b**) Density plots highlighting the expression and co-expression of *IGHD*, *LAG3*, and *PDCD1*. (**c**) Dotplot showing the top 15 differentially expressed genes in the B LAG3^+^ subpopulation. The dot size indicates the percent of expressing cells, and the dot color represents the normalized average expression. (**d**) Enrichment pathways analysis of B LAG3^+^ subpopulation using Reactome database. The size of each circle represents the number of genes belonging to a given pathway and such circles are colored by p-adjust. Kaplan–Meier curves for (**e**) Progression-free Interval (PFI), and (**f**) Overall Survival (OS) for the TNBC patients from the TCGA cohort. The patients were divided into Low-B LAG3^+^ and High-B LAG3^+^ groups using the *surv_cutpoints()* function. P-values were calculated using the omnibus MDIR test, which combines log-rank, Mantel–Haenszel, and Tarone-Ware test for the non-proportional hazards.

Given the observed increase of LAG-3 + B cells within the tumor microenvironment, along with their notable proliferation in the tumor-draining lymph nodes (tDLNs) of 4T1 and E0771 tumor-bearing mice, we proceeded to investigate the prevalence and potential functional roles of these cells in human breast cancer patients, as well as their broader implications for tumor biology and patient outcomes. To achieve this objective, we assessed single-cell RNAseq integrated data comprising tumor-derived cellular populations from breast cancer patients. After selecting B cells by evaluating the expression of canonical cellular markers (CD79A/B, MS4A1, and MBZ1), we evaluated highly variable genes among these cells, and separating these cells according to distinct genes expression as it follows: naïve B cells (CD19, MS4A1, IGHD, IGHM), memory B cells (CD19, MS4A1, CD27), plasma cells (IGHG1, IGHG2, MZB1, CD38), proliferative (CD19, MSA1, CD14), and LAG-3 + (CD19, LAG-3) (Fig. 5a). Although LAG-3 B cells comprised a minor portion of total tumor-infiltrating B cells (49 cells), evaluating other cellular markers, we observed co-expression of IGHD and PDCD1 alongside LAG-3 (Fig. 5b). Within the LAG3 + B cell subgroup, we observed elevated expression of FCRL4, HCK, CCR1, ITGAX, IFIT1, and in minor extent OSTN-AS1, SOX5, IFIT1, ADAP1, PDCD1, GPR25, ZEB2-A21, RBPMS2, FAM72B, EIF3P3 (Fig. 5c). Moreover, through enrichment pathway analysis using Reactome database, we observed that these cells had enrichment for costimulatory CD28 receptor family as well as for IFN-ɣ, and IFN-α/β pathways (Fig. 5d). Interestingly, we observed that TNBC patients that presented enrichment for LAG-3 + B cells transcripts had a significant better progression-free interval (PFI) rate compared to those patients with lower enrichment levels (Fig. 5e). Despite that, we could not observe difference in the overall survival (OS) rate between patients with low and high LAG-3 B cell transcripts, although we observed a tendency for better OS among patients with higher LAG-3 + B cell enrichment (Fig. 5f).

## Discussion

Diagnosed in younger women and known for their aggressive behavior, triple-negative tumors (TNBC) have posed a challenge to the medical field in the last decades. Interestingly, despite being associated with a worse prognosis, triple-negative tumors are more likely to present intense lymphocyte infiltrate, a finding that has been correlated with better overall survival rates and better response to chemotherapy^44,45^. Though most studies demonstrate that on average T cells are the most prominent lymphocyte population in breast tumors, other cellular profiles including B cells are also described, yet how tumors affect these populations remains largely unknown^46^. Here, using 4T1 and E0771 breast cancer murine models we initially demonstrated that these tumors presented a distinct kinetics profile despite mice being injected with the same number of cells. While the larger volume and detection of necrotic areas in 4T1 induced tumors are suggestive of a more pronounced proliferation by these cells compared the E0771 cell line, other variables are likely involved.

While both cell lines mimic the behavior of human breast tumors, 4T1 cells have are considered a model for stage IV breast cancer, while E0771 cells staging is unknown^47,48^. Although the classification of murine cell lines and their relation to human tumors may be difficult to establish, evidence demonstrates that this cell line resembles the basal-like intrinsic subtype, as authors pointed for the expression of cytokeratin-14, as well as high levels of proliferation and metastasis markers, such as Top2a, Birc5, Msln, Plk1^48^. Though the E0771 has also been isolated from a spontaneous tumor from the C57BL/6 mice strain, the cell line features distinct morphology and different enrichment cellular pathways. While the ongoing debate over the classification of E0771 tumors, transcriptome analysis demonstrates that these cells share common features with claudin-low tumors, with enriched pathways for angiogenesis, invasion, and epithelial-to-mesenchymal transition properties. Additionally, it has been shown that E0771 cells have lower differentiation and proliferation profiles^47,49^. Despite not evaluating the effects of tumor development on B cell populations, authors also demonstrated that 4T1 tumors were larger compared to E0771 after each correspondent period of analysis, further corroborating with the distinction between the two cell lines^50–52^.

The difference between the basal-like (4T1) and claudin-low (E0771) murine models was also observed when we analyzed the effect of tumor development in SLOs. Although the elevation of myeloid cells in the spleen, as well as lymph nodes are recognized in 4T1 tumor-bearing mice—and it was reported by our group and previous authors^53–55^, few studies have evaluated the kinetics of these cells in non-basal-like breast tumors. While different authors demonstrate that obesity, as well as distinct treatment regimens including immune checkpoint blockage (anti-PD-1)^56^, gemcitabine^57^, and ranitidine^58^ affects the frequency of myeloid CD11b + populations in the TME and spleen of E0771 tumor-bearing mice, timepoints were limited, preventing observing the potential alterations throughout tumor development. In the current study, despite detecting Ly-6C + Ly-6G- and Ly-6C^low/-^ Ly-6G + cells in the spleen of E0771 tumor-bearing mice, we did not observe major alterations throughout tumor growth. This could explain the lack of morphologic alteration in the spleen of E0771-injected mice, since continuous elevation of myeloid cells majorly contribute to the enlargement of the organ. Collectively, these findings lead us to believe that G- and GM-CSF production by 4T1 cells is more pronounced in comparison to E0771 cells. Corroborating this hypothesis, despite not evaluating the concentration of G-CSF throughout tumor development, Hiraga and colleagues observed elevated plasma levels of G-CSF in mice inoculated with 4T1 compared to E0771^59^. In addition to these results, while not evaluating E0771 cell line, authors demonstrated higher concentration of G-CSF from 4T1-derived cell culture supernatants compared to other murine cell lines^60^.

Interestingly, in addition to presenting distinct kinetics for myeloid-derived cells throughout 4T1 and E0771 tumor development, a distinct dynamic for the lymphoid cell compartment was also observed in the spleen. Despite presenting an increased frequency and absolute cell count for B and T cells in later periods (21 days), this increase was more pronounced in E0771 tumor-bearing. Considering recent findings demonstrating that MDSCs cell are able to hamper B cell expansion and differentiation through the suppression of Tfh cells, as well as production of ROS, arginase-1 and nitric oxide (NO)^61–63^, it may be possible that expanded MDSCs in the spleen of 4T1 tumor-bearing mice difficult the expansion of local B cells. In addition to these differences, we only demonstrated elevated frequency of IgD^int-low^ B cell in the spleen of E0771 tumor-bearing, further suggesting for the negative impact of MDSCs in the differentiation process of B cells in the spleen of 4T1 tumor-bearing mice. Intriguingly, while decreased expression of IgD in B cells indicates an ongoing differentiation process and isotype switch, the elevation in IgD^int-low^ B cell in the spleen of E0771 model was not followed by a correspondent increase of plasma cells (CD138 +). While we have evaluated antibody-secreting cells, it may be possible that emerging B cells have memory phenotype (CD27 +). Although the current paper highlights that kinetics of splenic B cell subpopulations throughout claudin-low and basal-like breast tumors growth, future studies are necessary to address other B cell subpopulations, as well as understanding the potential differences in antigens derived from distinct breast cancer subtypes.

Despite CD11b + myeloid cells were also detected in tDLNs in both murine models, these populations comprised a minor fraction of total leukocytes (< 1%), with B cells accounting for most of the cells following the tumoral challenge. In opposition to the spleen which only has access to antigens transported through the blood, tDLNs and associated lymphatic vessels are located adjacent to the tumor site, and provide immediate access to TAAs shed from eliminated and necrotic tumor cells^64^. These differences may explain the early elevation of total B cells in tDLNs in both murine models in comparison to the spleen. Furthermore, as previously demonstrated, MDSCs (CD11b + Ly-6G + Ly-6Clow-) were almost absent in LNs. In a recent study, authors demonstrated that tDLNs from E0771 tumor-bearing mice undergo an intense morphological change following tumoral implantation. According to Louie et al. tumor growth leads to the expansion of LN conduits in the B cell zone and disruption of the sub-capsular sinus (SCS) macrophage layer, favoring the transport of TAAs to GC zones and associated follicular dendritic cells (FDCs)^65^. Despite not assessing the kinetics of distinct B cell subsets through the weeks of tumor development, Louie et al. reported the appearance of GC B cells in the tDLNs of the E0771 model as early as 7 days post-tumor implantation, indicating activation and proliferation of B cells in this site. Corroborating these findings, Gu Y. et al*.* analyzing the role of B cells in the formation of lymph node metastasis, reported an increase in both cell count and frequency of CD19 + B220 + cells in the tDLN of 4T1 tumor-bearing mice. Additionally, the authors also demonstrated that this elevation was more pronounced in tDLNs compared to non-draining lymph nodes (nDLN)^66^. The capacity of B cells to produce antibodies against tumoral antigens was confirmed in the work of Díaz-Zaragoza and colleagues, that demonstrated serum-derived IgM antibodies capable of recognizing 4T1 protein extracts^67^.

In agreement with these results, we reported a significant elevation in total B cells, as well as B LAG-3 + cells and plasma cells in the tDLNs not only 7 days after the initial tumor injection, but a sustained proliferation throughout the weeks, indicating a continuous influx of TAAs to tDLNs, leading to activation, proliferation and differentiation of B cells, that may or may not migrate to the TME. The relevance of tDLNs in priming humoral immune response against tumor antigens was observed in another study, in which the transferring of activated tDLN B cells resulted in the inhibition of lung metastasis. Moreover, the combined B and T cell transfer enhanced T cell response against 4T1 tumors^68^. Though collectively these findings support the elaboration of a humoral immune response against TAAs and TSAs, it may not be excluded an unspecific elevation of lymphocytes in these organs due to tumor-derived cytokines. Previous works demonstrated that both 4T1 and E0771 cell lines promote the elevation of pro-inflammatory cytokines (e.g. IFN-γ, TNF, IL-1β, IL-6, GM-CSF), which could contribute to elevate lymphocytes in SLOs^69,70^.

Although different studies in pre-clinical and clinical settings have reported the presence of B cells in the TME^71^, the impact of earlier tumor development in B cell kinetics is still unknown. Interestingly, both 4T1 and E0771 tumor development caused infiltration of B cells in the TME as early as 7 days of implantation, yet changes were less pronounced compared to SLOs, and most of these populations were comprised of IgD + cells. These results contrast with those observed in the spleen and especially in the tDLNs, which demonstrated an elevation in plasma cells (CD138 +) in addition to the IgD + B cells. These results suggest that although B cells may be able to migrate to the TME, proliferation, and differentiation into antibody-secreting stages may be hampered, either by the immune suppression exerted by distinct tumor-infiltrating cell profiles (Tregs, MDSCs)^61–63^, and/or absence of essential cellular and molecular components required for the optimal B cell activation and selection, such as cognate activation by T cells, and co-stimulatory signaling from Tfh cells^72^. Corroborating this hypothesis, one study demonstrated that although E0771 tumors were followed by expanded tumoral vasculature, there was a progressive decrease of infiltrating B lymphocytes alongside an increase of MDSCs and Tregs cells^73^.

Although the detection of B cells in the TME is suggestive of an ongoing anti-tumoral response, and recent studies using the 4T1 murine model propose that tDLN B cells are capable of mediating tumor cell death through Fas-FasL interaction^74^, the maintenance of immune suppression is considered one of the cancer hallmark^75^. Despite lacking a proper lineage marker similar to FOXP3 in Tregs, it has been demonstrated B regulatory cells can contribute to immune suppression by implementing distinct mechanisms, including production of IL-10, IL-35, TGF-β and granzyme B (GZMB)^76,77^. Although distinct Bregs populations, such as CD25^+^CD81^High^ B7H-1^High^, PD-1- PD-L1 + CD19 + , and CD19^+^ CD25^High^ CD69^High^ have been described for breast cancer murine models^30,78, 79^, most studies use a single time point, preventing understand how tumor development influence these cells. Moreover, despite the extensively used 4T1 model (basal-like), it’s important to better understand the dynamic of Bregs in other breast tumor subtypes, as the emerging results could result in a better overall comprehension of the existing diverse breast malignant neoplasias. In an attempt to better understand the emergence of Breg phenotypes in the early moments of carcinogenesis, we used two distinct murine models. In this regard, considering the distinction between 4T1 and E0771 cell lines, we hypothesized that basal-like and claudin-low tumor development could lead to distinct increase in Bregs phenotypes in SLOs, but, more importantly, in the TME. Curiously, although Bregs populations have been implicated in pro-tumoral action in pancreatic adenocarcinoma^80^, and lymphoma^81^, in the current study we were not able to detect B10 Breg (CD5 + CD1d +) phenotype in the TME of neither the used murine models, suggesting that other mechanisms may be involved in the emergence of this phenotype other than differences in the breast tumor subtype. Although others have demonstrated the existence of B10 B cells in the spleen, peritoneal cavity and lymph nodes this cellular phenotype comprises a minor portion among total B cells, comprehending up to 8% in peripheral blood, and 4% in mesenteric lymph node^82,83^. Though challenging, we detected this phenotype in the spleen and tDLN of both murine models, 4T1 and E0771 tumor development did not cause alterations in the frequency of these cells. Despite these results, the existence of B10 Bregs in the TME should not be excluded, since the phenotype comprises only a minor fraction of total B cells (< 0.3%).

Interestingly, although B10 Breg cells remained unaltered in SLOs and were absent in the TME, in the present work we detected LAG-3 + B cells in all the assessed tissues and timepoints (7-, 14- and 21-days post tumor cell injection). In addition, we reported an elevation of the absolute cell count for LAG-3 + B cells in the tDLNs of both murine models throughout the weeks. In opposition to the previous results, here we observed a more pronounced increase of this phenotype in the 4T1 tumor-bearing mice, which could suggest that distinct breast tumor subtypes may distinctively influence the emergence of LAG-3 + B cells. Yet, similar to B10 Breg cells, LAG-3 + B cells comprised a minor fraction of total B cells, accounting for up to 2 and 3% of B cells in the spleen and tDLNs, respectively. Although this immune checkpoint molecule was initially characterized in T cells^84^, it has also been described in B cells, yet the role in B cells is still elusive, as different ligands have been proposed, and the downstream signaling pathway is still unknown^85^. Early findings from in vitro experiments demonstrated that the expression of LAG-3 can be a T-dependent activation marker in B cells^86^. Considering the capacity of tumor cells to promote sustained expression of IC, we hypothesized that tumor growth could impact the expression of LAG-3 in B-TILs, yet we only observed a transient elevation in 4T1 tumors. While the causes of this transient elevation need better clarification in future experiments, it may be possible that 14-day tumor growth corresponds to the period of higher B cell activity in these tumors, followed by suppression of TILs and predominance in MDSCs as previously demonstrated in a study^54^. In case this hypothesis is correct, the absent increase of LAG-3 + B cells in the E0771 TME, could either indicate that B cells are not properly stimulated in that microenvironment, or that B cells reach peak stimulation in a period beyond the last timepoint (21 days). Interestingly, despite their distinct classification and differences in the kinetics of LAG-3 + B cells, both 4T1 and E0771 tumor-bearing mice presented a similar composition when analyzing different cell markers within the LAG-3 + B cell compartment. We observed that most of these cells had high expression of IgD + cells, suggesting that these are early activated B cell stages, rather than another Breg phenotype. Curiously, despite detecting this phenotype in non-tumoral conditions, only in the tumor-bearing mice a cell count elevation in tDLNs was observed. Considering the IgD and LAG-3 co-expression in these cells, LAG-3 may provide inhibitory signaling to balance activation of BCR after antigen binding and recognition, in a similar manner to T cells, which counterbalances TCR activation by inhibiting calcium flux and activation of nuclear factor of activated T cells (NFAT)^87^. Despite naturally LAG-3 + CD138^hi^ regulatory B cell subset has been reported in one study, and authors demonstrated that the population may emerge from B1b and other B cell subsets in a BCR-dependent manner, our study did not identify this cell profile in SLOs and TME throughout time, suggesting that these cells may appear under specific pathological conditions.

Although our initial analysis corroborates with the hypothesis of LAG-3 B cells being early-activated stages, as these cells also had IgD transcripts, we also observed genes that are involved in other cellular stages. Belonging to the Fc receptor-like family, studies demonstrate that FCRL4 is mainly expressed in memory B cells in sub-epithelial regions, and the presence of the receptor is associated with viral infections (e.g. HIV) and autoimmune diseases (e.g. rheumatoid arthritis), yet authors demonstrate that other cellular stages might express this receptor, including plasma cells^88,89^. Authors argue that FCRL4 is involved in pro-inflammatory B cell stages, contributing to balancing the BCR response, as FCRL4 activation leads to phosphorylation of ITIM domains by scr-family kinases, ultimately contributing to a lower cell cycle in B cells. Interestingly, studies demonstrate that other genes and proteins are associated with FCRL4 expression, including surface receptors (ITGAX (CD11c)), chemokine receptors (CCR1, CCR5), scr-family kinases (HCK, LYN) transcription factors, as well as activation-induced cytidine deaminase (AICDA)^89–92^ – some of which have been detected in our analysis. Although other genes (e.g. ADAP1, OSTN-AS1, ZEB2-A21) were found differentially expressed in LAG-3 B cells, their role and contribution to this phenotype are still elusive, yet authors demonstrate that ZEB2 alongside ITGAX is essential to B cell differentiation^93,94^.

Interestingly, we also observed expression of PDCD1, despite the immune checkpoint receptor being known to balance activation of T cells by modulating MHC-TCR interaction, evidence suggests that this receptor is also involved in balancing BCR activation signaling^95,96^. Additionally, the enriched CD28 costimulatory pathway in LAG-3 + B cells further suggests the involvement of these cells with activated B cell stages, as ligands of CD28 (CD80/CD86) are found in activated B cells, and are associated with germinal center B survival^97,98^. Though the functions of LAG-3 B cells need better clarification, our study suggests that these cells may be associated with a good prognosis. Although we could not demonstrate distinction in the OS rate between patients with high and low LAG-3 B cell transcripts, we observed a tendency for better overall survival among patients with higher enrichment. More importantly, we reported that patients with elevated LAG-3 B cell transcripts had a better progression-free interval compared to patients with lower transcript levels. Collectively, these results suggest that LAG-3 B cell subset is most likely involved in proliferative B cell stages, associated with the emergence and differentiation of memory and plasma cells, and elevated presence could indicate a more robust humoral immune response in part of the TNBC patients. Despite the potential relevance of these populations for breast tumor patients, limitations should be noted. First, although B cells may be found in the TME, these cells comprise minor portion of TILs, and subpopulations (e.g. LAG-3 + B cells) are rarer, making it difficult to evaluate. Second, despite assessing high quality single-cell datasets, future analysis should take in consideration larger patient cohorts to validate the current findings. Third, although we investigated differentially expressed genes and pathways in TIL LAG-3 + B cells, isolation and a more detailed transcriptome characterization should be helpful in better understanding these cells in both physiological and pathological contexts.

Here, using two distinct murine syngeneic models, we demonstrated that the development of basal-like (4T1) and claudin-low (E0771) murine breast cancer models have distinct implications in the dynamic of myeloid-derived and lymphocytic-derived cells in the SLOs and TME. While the elevation of myeloid-derived cells is well recognized in the basal-like 4T1-induced tumors, here we demonstrated that while present, E0771-induced tumors do not cause expansion of these cells in SLOs. More importantly, the distinction between the two murine models was also observed in evaluating B cell subpopulations. While both induced tumors caused an elevation of B cells in tDLNs as early as 7 days post tumor cell inoculation, in addition to the rapid expansion in the organ area, 4T1-induced tumors caused a more robust elevation of IgD^high^, IgD^Int-low^, and CD138 + cells compared to E0771 induced tumors. Moreover, despite the emergence of distinct Breg subtypes in malignant neoplasias, to the best of our knowledge, here we reported for the first time the dynamic of B10 and, most importantly, LAG-3 + B cells under physiological, basal-like, and claudin-low breast cancer subtypes. Although the functions associated with LAG-3 receptor in B cells remain to be elucidated, here we demonstrated that rather than just a regulatory marker, the receptor is most likely associated with a subset of activated B cells. Corroborating with this hypothesis we demonstrated the expression of relevant cell cycle and proliferation genes alongside LAG-3. Despite facing challenges such as restricted time points for analysis and limited sample yields from tumors, this study underscores the distinct B cell dynamics and the development of humoral responses during early carcinogenic events in basal-like and claudin-low breast cancer subtypes. It also sheds light on the potential roles of LAG-3 + B cells within the context of Triple-Negative Breast Cancer (TNBC). We advocate for further research and experimental studies to deepen our understanding of the specific functions and mechanisms of action of these B cell phenotypes across various breast cancer subtypes.

### Supplementary Information

Supplementary Figures.

## Data Availability

Data shall be available from the corresponding author upon request.

## Abbreviations

TNBC: Triple-negative breast cancer
ER: Estrogen receptor
PR: Progesterone receptor
HER: Human epidermal receptor
TILs: Tumor-infiltrating lymphocytes
OS: Overall survival
IFN-y: Interferon gamma
IL-2: Interleukin 2
Treg: T regulatory cell
TME: Tumor microenvironment
DFS: Disease-free survival
RFS: Relapse-free survival
ASC: Antibody-secreting cell
Breg: B regulatory cell
MHC-II: Major histocompatibility complex II
LAG-3: Lymphocyte activation gene 3
IL-35: Interleukin 35
TGF-b: Transforming growth factor beta
tDLN: Tumor-draining lymph node
PBS: Phosphate-buffered saline
PMN: Polymorphonuclear
MN: Mononuclear
tSNE: T-distributed stochastic neighbor embedding
TSAs: Tumor-specific antigens
TAAs: Tumor-associated antigens
GC: Germinal center
GZMB: Granzyme B
NFAT: Nuclear factor of activated T-cells
SLOs: Secondary lymphoid organs
