# Supplementary material for: Dynamic changes in B cell subpopulations in response to triple-negative breast cancer development
Source: Sci Rep. 2024 May 21;14:11576. doi: 10.1038/s41598-024-60243-y (PMC11109097; doi:10.1038/s41598-024-60243-y)
Supplement: Supplementary file 1 — Supplementary Figures. [file 41598_2024_60243_MOESM1_ESM.docx]

**Supplementary Material**

**
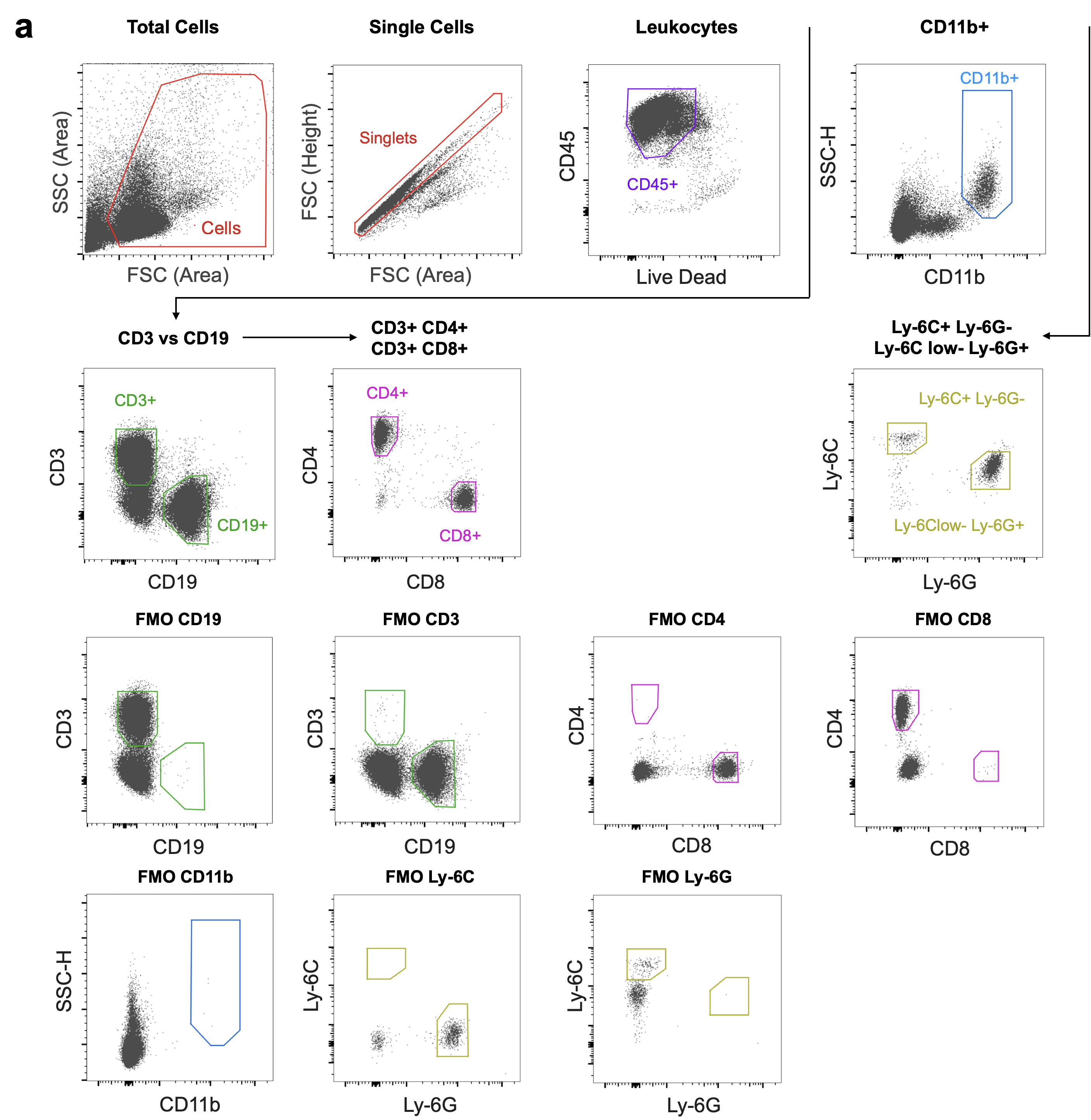
**


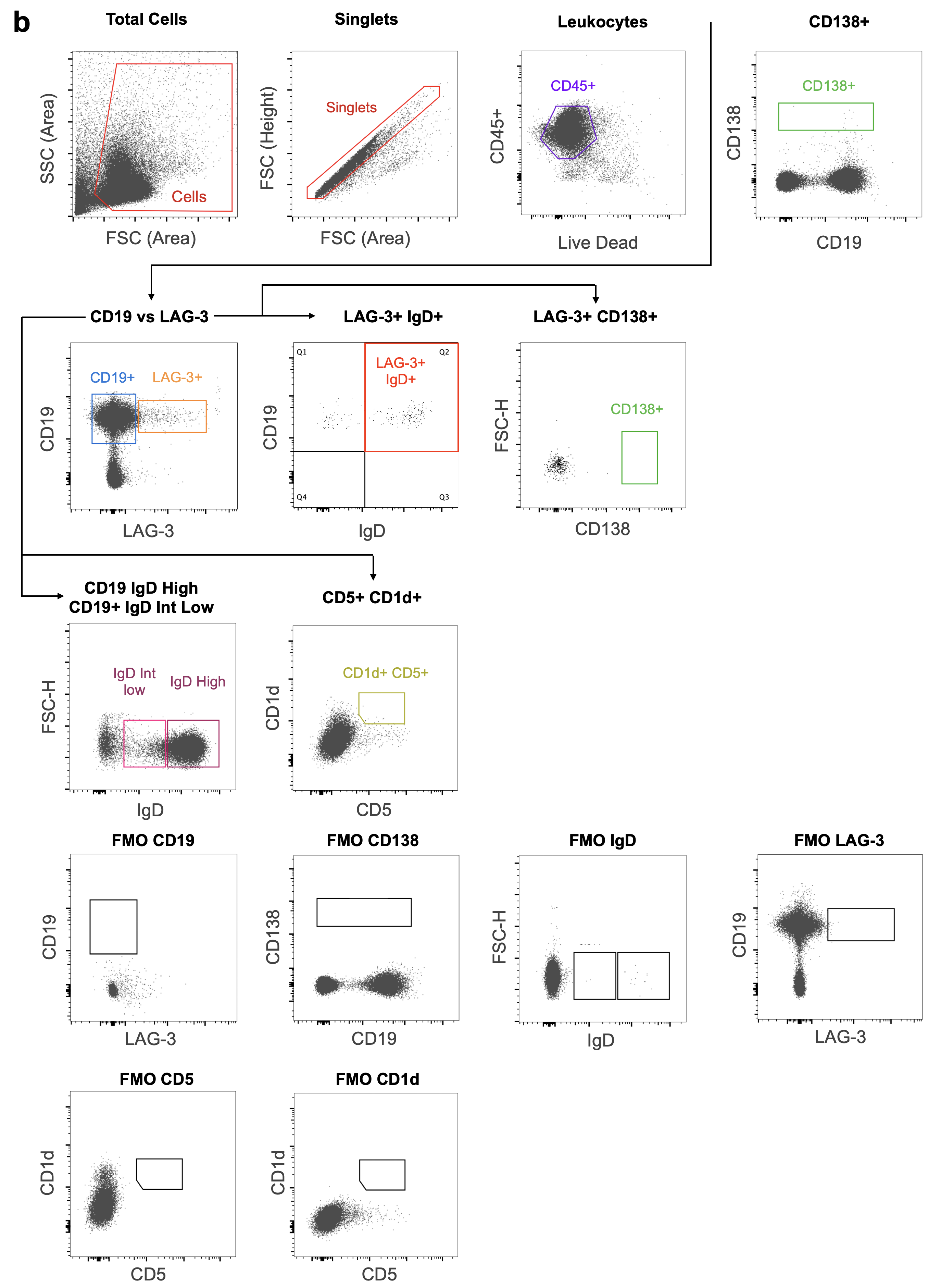


**Figure S1.** **Gating strategies for the assessment of myeloid and lymphoid cells.** Total cells were gated by plotting forward scatter area (FSC-A) versus side scatter area (SSC-A). Single cells were selected by plotting forward scatter area (FSC-A) versus forward scatter height. Live cells were selected by gating unstained cells for the viability dye. Total leukocytes in the samples were selected by gating CD45+ cells. **(a)** Lymphoid cell populations were gated by plotting CD19 versus CD3, and myeloid cells were gated by plotting side scatter height (SSC-H) versus CD11b. Each population of interest were gated as follows: B Cell (CD45+ CD19+); TCD4 Cells (CD45+ CD3+ CD4+); TCD8 Cells (CD45+ CD3+ CD8+); Polymorphonuclear myeloid cells (CD11b+ Ly-6G+ Ly-6C-); Mononuclear myeloid cells (CD11b+ Ly-6G- Ly-6C+). **(b)** Each population of interest were gated as follows: B Cell (CD45+ CD19+); Plasma cells (CD45+ CD138+); B10 B cell (CD45+ CD19+ CD5+ CD1d+), Naive B Cell (CD45+ CD19+ IgD High), Early-activated B cells (CD45+ CD19+ IgD Int Low); B Cell LAG-3+ subsets (CD45+ CD19+ LAG-3+): IgD Int Low, IgD High, CD138+.


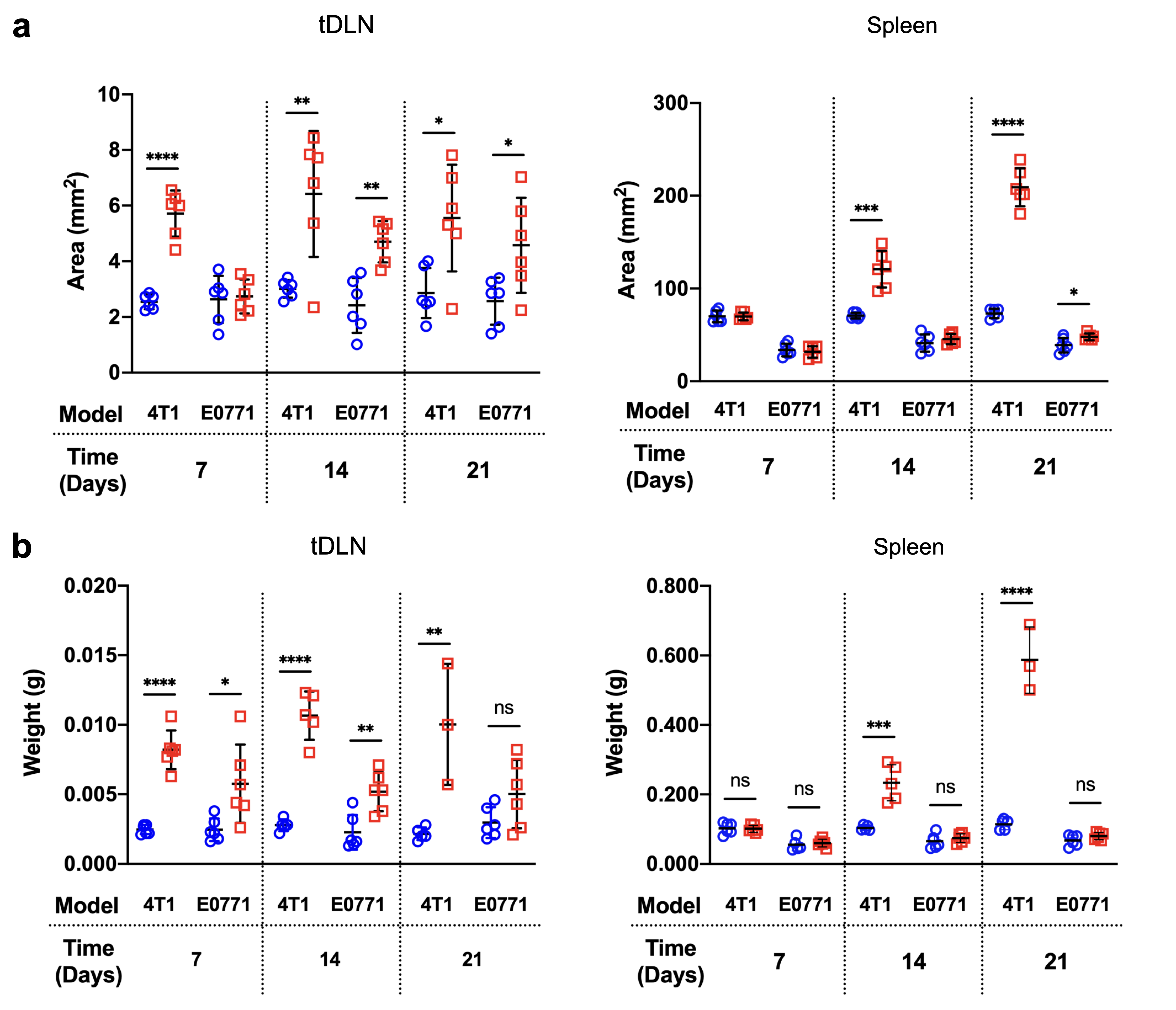


**Figure S2. Area and weight of spleen and tDLNs.** Area of the tDLNs (right) and the spleen (right) in mice from control (blue) and 4T1/E0771 tumor-bearing mice after 7, 14 and 21 days of tumoral challenge **(a).** Weight of the tDLNs (right) and the spleen (right) in mice from control (blue) and 4T1/E0771 tumor-bearing mice after 7, 14 and 21 days of tumoral challenge **(b).** Student t-test. p<0.05, ** p<0.01, *** p<0.001, **** p<0.0001.


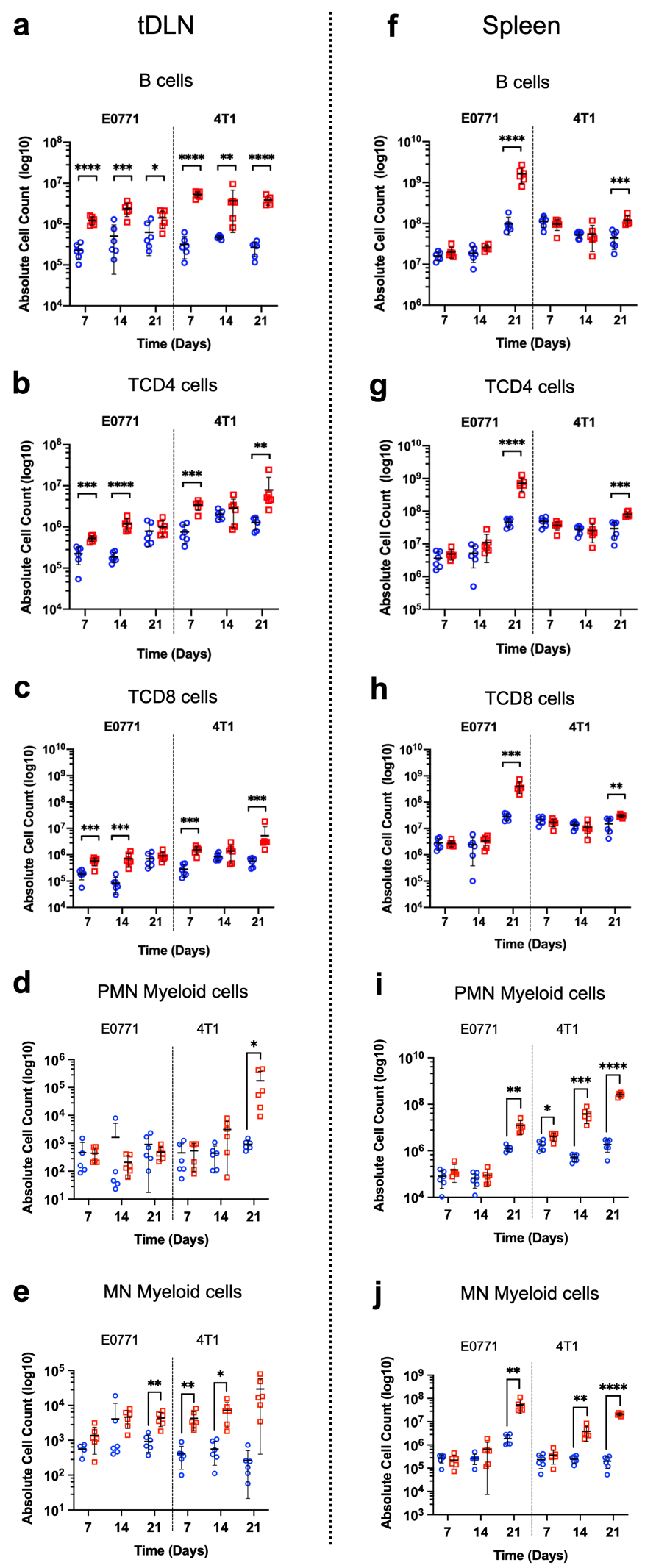


**Figure S3. Absolute cell count for lymphoid and myeloid cells in the tDLN and spleen.** Cell count for B cells **(a, f)**, TCD4 cells **(b, g)**, TCD8 cells **(c, h)**, PMN myeloid cells **(d, i),** and MN myeloid cells **(e, j)** in the tDLNs and the spleen from control (blue) and 4T1/E0771 tumor-bearing mice (red) 7, 14 and 21 days after the initial inoculation, respectively**.** Student t-test. p<0.05, ** p<0.01, *** p<0.001, **** p<0.0001.


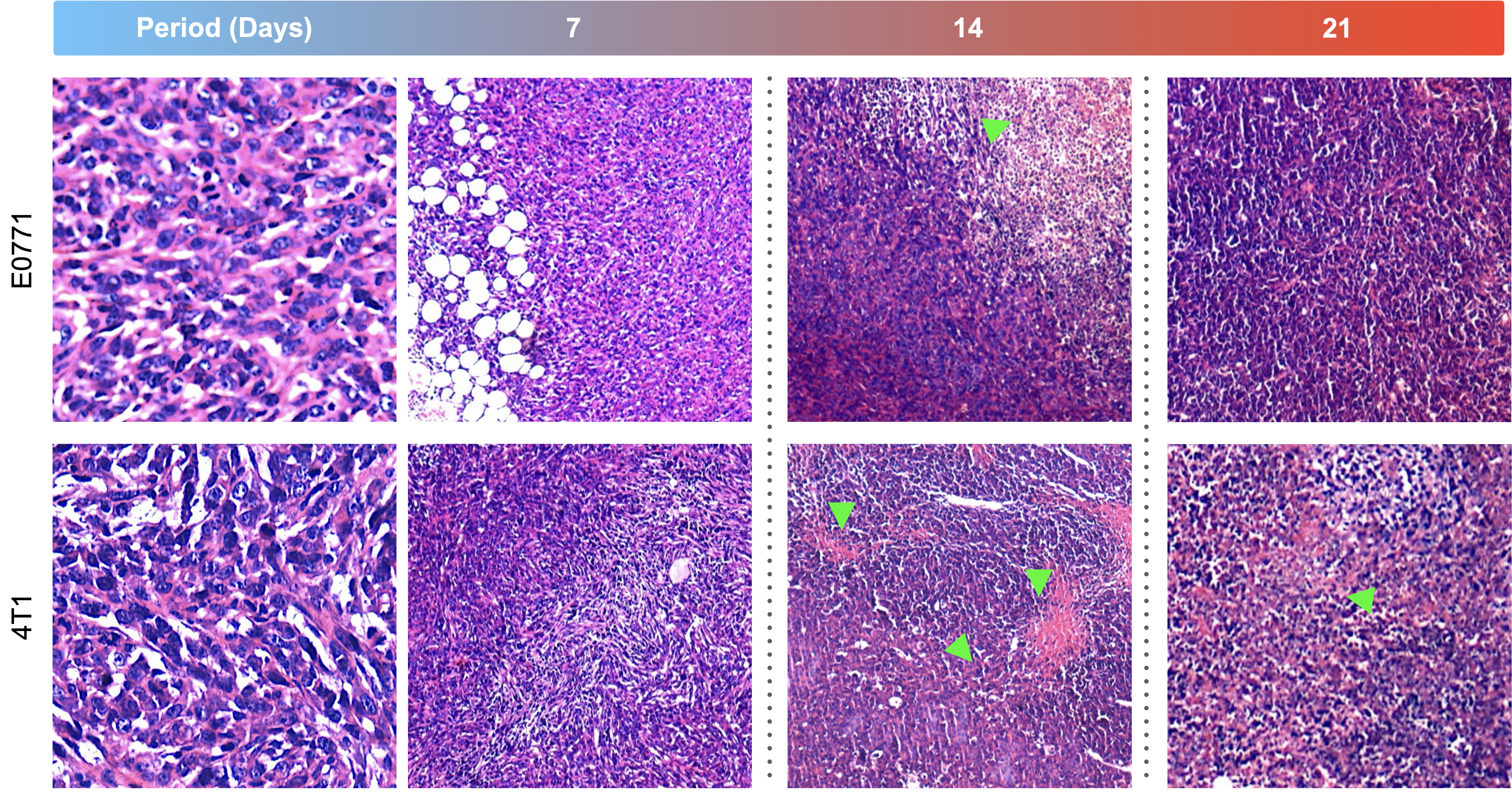


**Figure S4. Histopathological evaluation of 4T1 and E0771 induced tumors.** Representative image of 4T1 (upper row) and E0771 (bottom row) induced tumors after 7, 14 and 21 days of development. Tumor slides stained in Hematoxylin and Eosin (H&E) and analyzed under light microscopy (column 1: 40x magnification, columns 2-4: 4x magnification) Green arrows indicating necrotic areas.
